# Supplementary material for: Integrated methylome and phenome study of the circulating proteome reveals markers pertinent to brain health
Source: Nat Commun. 2022 Aug 9;13:4670. doi: 10.1038/s41467-022-32319-8 (PMC9363452; doi:10.1038/s41467-022-32319-8)
Supplement: Supplementary file 3 — Description of Additional Supplementary Files [file 41467_2022_32319_MOESM3_ESM.docx]

**Description of Additional Supplementary Files**

File Name: Supplementary Data 1

Description: Annotation information for the 4,058 protein levels (corresponding to 4,235 SOMAmer measurements) included in the study.

File Name: Supplementary Data 2

Description: Demographic and phenotypic information for the two Generation Scotland sample groups with protein level data that were used in analyses.

File Name: Supplementary Data 3

Description: Principal components analyses of 4,235 SOMAmer levels in the Generation Scotland (N=1,065) sample.

File Name: Supplementary Data 4

Description: 238,245 pQTMs (2,107 cis, 236,138 trans) identified in the basic methylome-wide association studies of 4,058 protein levels (corresponding to 4,235 SOMAmer measurements) in 774 individuals from Generation Scotland.

File Name: Supplementary Data 5

Description: 3,213 pQTMs (453 cis, 2,760 trans) identified in the estimated White Blood Cell proportion-adjusted methylome-wide association studies of 4,058 protein levels (corresponding to 4,235 SOMAmer measurements) in 774 individuals from Generation Scotland.

File Name: Supplementary Data 6

Description: 2,928 pQTMs (451 cis, 2,477 trans) with P < 4.5x10-10 in the fully-adjusted methylome-wide association studies of 4,058 protein levels (corresponding to 4,235 SOMAmer measurements) in 774 individuals from Generation Scotland.

File Name: Supplementary Data 7

Description: Lambda values for the 195 SOMAmer levels (corresponding to 191 unique proteins) that comprised the 2,928 pQTM associations in the fully-adjusted MWAS models.

File Name: Supplementary Data 8

Description: Summary of protein levels with pQTMs in the fully-adjusted EWAS with known pQTLs available for adjustment summarised for each.

File Name: Supplementary Data 9

Description: Number of pQTM associations in the fully-adjusted MWAS for each of the 195 SOMAmer levels (corresponding to 191 unique protein levels).

File Name: Supplementary Data 10

Description: Summary of the 1,837 unique CpGs with pQTMs that had P < 4.5x10-10 in the fully-adjusted MWAS.

File Name: Supplementary Data 11

Description: Replication assessment of 98 pQTMs reported by Zaghlool et al, 2020.

File Name: Supplementary Data 12

Description: The proteomic signature of age and sex in the Generation Scotland (n=1,065) sample.

File Name: Supplementary Data 13

Description: Brain imaging marker associations with circulating plasma proteins in the Generation Scotland (maximum n=1,065) sample.

File Name: Supplementary Data 14

Description: Cognitive score associations with circulating plasma proteins in the Generation Scotland (maximum N=1,065) sample.

File Name: Supplementary Data 15

Description: Associations between protein 4,235 SOMAmers and APOE e4 status in the Generation Scotland (N=1,065) sample.

File Name: Supplementary Data 16

Description: Summary of the 191 unique protein that were implicated in a total of 405 associations with neurological phenotypes.

File Name: Supplementary Data 17

Description: The 405 associations between protein levels and brain health outcomes that had P<3.5x10-4 in Generation Scotland (maximum N=1,065).

File Name: Supplementary Data 18

Description: Protein markers that were associated with both a cognitive score and a brain imaging phenotype in Generation Scotland (maximum N=1,065).

File Name: Supplementary Data 19

Description: Replication assessment for associations with cognitive scores and brain imaging traits.

File Name: Supplementary Data 20

Description: 35 CpG-protein (pQTM) associations involving proteins associated with either brain imaging, cognitive scoring or APOE status in Generation Scotland.

File Name: Supplementary Data 21

Description: ENCODE accession identifiers that were used to ascertain ChIP-seq data from peripheral blood mononuclear cells (PBMCs) and brain hippocampus (Brain).
