# Supplementary material for: Integrated methylome and phenome study of the circulating proteome reveals markers pertinent to brain health
Source: Nat Commun. 2022 Aug 9;13:4670. doi: 10.1038/s41467-022-32319-8 (PMC9363452; doi:10.1038/s41467-022-32319-8)
Supplement: Supplementary file 25 — Reporting Summary [file 41467_2022_32319_MOESM25_ESM.pdf]

Corresponding author(s): Danni A Gadd and Riccardo E Marioni

Last updated by author(s): Jul 13, 2022

Reporting Summary

Nature Research wishes to improve the reproducibility of the work that we publish. This form provides structure for consistency and transparency in reporting. For further information on Nature Research policies, see our Editorial Policies and the Editorial Policy Checklist.

Statistics

For all statistical analyses, confirm that the following items are present in the figure legend, table legend, main text, or Methods section.

n/a	Confirmed	
☐	☒	The exact sample size (*n*) for each experimental group/condition, given as a discrete number and unit of measurement
☐	☒	A statement on whether measurements were taken from distinct samples or whether the same sample was measured repeatedly
☐	☒	The statistical test(s) used AND whether they are one- or two-sided<br>*Only common tests should be described solely by name; describe more complex techniques in the Methods section.*
☐	☒	A description of all covariates tested
☐	☒	A description of any assumptions or corrections, such as tests of normality and adjustment for multiple comparisons
☐	☒	A full description of the statistical parameters including central tendency (e.g. means) or other basic estimates (e.g. regression coefficient) AND variation (e.g. standard deviation) or associated estimates of uncertainty (e.g. confidence intervals)
☐	☒	For null hypothesis testing, the test statistic (e.g. *F*, *t*, *r*) with confidence intervals, effect sizes, degrees of freedom and *P* value noted<br>*Give P values as exact values whenever suitable.*
☒	☐	For Bayesian analysis, information on the choice of priors and Markov chain Monte Carlo settings
☐	☒	For hierarchical and complex designs, identification of the appropriate level for tests and full reporting of outcomes
☐	☒	Estimates of effect sizes (e.g. Cohen's *d*, Pearson's *r*), indicating how they were calculated

Our web collection on statistics for biologists contains articles on many of the points above.

Software and code

Policy information about availability of computer code

Data collection	No formal software was used to collect blood samples for protein and DNA methylation datasets.
Data analysis	All code used in the analyses for this work as written by the authors and is housed at: https://github.com/DanniGadd/Epigenome-and-phenome-wide-study-of-brain-health-outcomes. This is open access. All code was developed using R.<br><br>Software and R packages used in analyses were as follows:<br>R (Version 4.0)<br>brainageR R package (Version 2.1)<br>OSCA software (Version 0.41)<br>circlize R package (Version 0.4.12)<br>coxme R package (Version 2.2-16)<br>prcomp in the stats R package (Version 3.6.2)

For manuscripts utilizing custom algorithms or software that are central to the research but not yet described in published literature, software must be made available to editors and reviewers. We strongly encourage code deposition in a community repository (e.g. GitHub). See the Nature Research guidelines for submitting code & software for further information.

Data

Policy information about availability of data

All manuscripts must include a data availability statement. This statement should provide the following information, where applicable:

- Accession codes, unique identifiers, or web links for publicly available datasets
- A list of figures that have associated raw data
- A description of any restrictions on data availability

The fully-adjusted MWAS summary statistics for 4,231 protein levels generated in this study have been deposited in the MRC-IEU EWAS catalog. These files are also available through the Zenodo repository at https://doi.org/10.5281/zenodo.6801458. A YouTube video summarising the findings of the study and detailing how to access the summary statistics files can be viewed at https://www.youtube.com/channel/UCxQrFFTIItF25YKfJTXuumQ.
Datasets generated in this study are made available in Supplementary Data files 1-21. Source Data files 1-5 provide data used to plot Figs. 2c, 3a, 4a, 4b and 5. The raw data from Generation Scotland are not available due to them containing information that could compromise participant consent and confidentiality. Generation Scotland is run as a Resource for the research community. Requests to use the Resource are made from: Academic collaborators: employees who are party to the Generation Scotland Collaboration Agreement, or researchers or employees of an academic institution or the NHS. Commercial organisations: specific arrangements have been defined to allow commercial organisations to access Generation Scotland resources. Data can be obtained from the data owners. Instructions for accessing Generation Scotland data can be found here: https://www.ed.ac.uk/generation-scotland/for-researchers/access; the GS Access Request Form can be downloaded from this site. Completed request forms must be sent to access@generationscotland.org to be approved by the Generation Scotland Access Committee.
For any further correspondence and material requests please contact Dr Riccardo Marioni at riccardo.marioni@ed.ac.uk.

Field-specific reporting

Please select the one below that is the best fit for your research. If you are not sure, read the appropriate sections before making your selection.

☒ Life sciences ☐ Behavioural & social sciences ☐ Ecological, evolutionary & environmental sciences

For a reference copy of the document with all sections, see nature.com/documents/nr-reporting-summary-flat.pdf

Life sciences study design

All studies must disclose on these points even when the disclosure is negative.

Sample size	The datasets were chosen due to the availability of proteomic, epigenetic and phenotypic data. Maximum available samples were used in all cases.
Data exclusions	Participant data was only excluded where there were individuals who did not have matched samples available (i.e. epigenetic and proteomic data were available in a subset of the 1,065 individuals with protein levels measured). All other data were included.
Replication	Replication in an external cohort was not available in this study.
Randomization	N/A - No randomization was required. All participants were included and there were no experimental groups.
Blinding	N/A - This study did not include the allocation of experimental groups and profiled all possible associations between EpiScores and traits.

Reporting for specific materials, systems and methods

We require information from authors about some types of materials, experimental systems and methods used in many studies. Here, indicate whether each material, system or method listed is relevant to your study. If you are not sure if a list item applies to your research, read the appropriate section before selecting a response.

Materials & experimental systems

n/a	Involved in the study	
☒	☐	Antibodies
☒	☐	Eukaryotic cell lines
☒	☐	Palaeontology and archaeology
☒	☐	Animals and other organisms
☐	☒	Human research participants
☒	☐	Clinical data
☒	☐	Dual use research of concern

Methods

n/a	Involved in the study	
☒	☐	ChIP-seq
☒	☐	Flow cytometry
☒	☐	MRI-based neuroimaging

Human research participants

Policy information about studies involving human research participants

Population characteristics	Generation Scotland: the Scottish Family Health Study (GS) is a large, family-structured, population-based cohort study of >24,000 individuals from across Scotland. The Stratifying Resilience and Depression Longitudinally (STRADL) cohort is a subset of 1,188 individuals from the GS cohort who undertook additional assessments approximately five years after the study baseline.<br>Population demographics can be found in Supplementary Data 2 of this study, for each group used in the analyses.
Recruitment	Recruitment for Generation Scotland: the Scottish Family Health Study (GS) took place between 2006 and 2011 with a clinical visit where detailed health, cognitive, and lifestyle information was collected along with biological samples (blood, urine, saliva). The Stratifying Resilience and Depression Longitudinally (STRADL) cohort is a subset of 1,188 individuals from the GS cohort who undertook additional assessments approximately five years after the study baseline.
Ethics oversight	All components of GS received ethical approval from the NHS Tayside Committee on Medical Research Ethics (REC Reference Number: 05/S1401/89). GS has also been granted Research Tissue Bank status by the East of Scotland Research Ethics Service (REC Reference Number: 20/ES/0021), providing generic ethical approval for a wide range of uses within medical research. All participants included in the current study provided informed consent for the use of their data for biomedical research.

Note that full information on the approval of the study protocol must also be provided in the manuscript.
